# Assessment of the right ventricle with cardiovascular magnetic resonance at 7 Tesla

**DOI:** 10.1186/1532-429X-15-23

**Published:** 2013-03-14

**Authors:** Florian von Knobelsdorff-Brenkenhoff, Valeriy Tkachenko, Lukas Winter, Jan Rieger, Christof Thalhammer, Fabian Hezel, Andreas Graessl, Matthias A Dieringer, Thoralf Niendorf, Jeanette Schulz-Menger

**Affiliations:** 1 Berlin Ultrahigh Field Facility (B.U.F.F.), Max-Delbrueck Center for Molecular Medicine, Berlin, Germany; 2 Working Group on Cardiovascular Magnetic Resonance, Experimental and Clinical Research Center, a joint cooperation between the Charité Medical Faculty and the Max-Delbrueck Center for Molecular Medicine, HELIOS Klinikum Berlin Buch, Department of Cardiology and Nephrology, Berlin, Germany; 3 Experimental and Clinical Research Center, a joint cooperation between the Charité Medical Faculty and the Max-Delbrueck Center for Molecular Medicine, Berlin, Germany

**Keywords:** Magnetic resonance imaging, Right ventricle, Ultrahigh field, Cardiac

## Abstract

**Background:**

Functional and morphologic assessment of the right ventricle (RV) is of clinical importance. Cardiovascular magnetic resonance (CMR) at 1.5T has become gold standard for RV chamber quantification and assessment of even small wall motion abnormalities, but tissue analysis is still hampered by limited spatial resolution. CMR at 7T promises increased resolution, but is technically challenging. We examined the feasibility of cine imaging at 7T to assess the RV.

**Methods:**

Nine healthy volunteers underwent CMR at 7T using a 16-element TX/RX coil and acoustic cardiac gating. 1.5T served as gold standard. At 1.5T, steady-state free-precession (SSFP) cine imaging with voxel size (1.2x1.2x6) mm^3^ was used; at 7T, fast gradient echo (FGRE) with voxel size (1.2x1.2x6) mm^3^ and (1.3x1.3x4) mm^3^ were applied. RV dimensions (RVEDV, RVESV), RV mass (RVM) and RV function (RVEF) were quantified in transverse slices. Overall image quality, image contrast and image homogeneity were assessed in transverse and sagittal views.

**Results:**

All scans provided diagnostic image quality. Overall image quality and image contrast of transverse RV views were rated equally for SSFP at 1.5T and FGRE at 7T with voxel size (1.3x1.3x4)mm^3^. FGRE at 7T provided significantly lower image homogeneity compared to SSFP at 1.5T. RVEDV, RVESV, RVEF and RVM did not differ significantly and agreed close between SSFP at 1.5T and FGRE at 7T (p=0.5850; p=0.5462; p=0.2789; p=0.0743). FGRE at 7T with voxel size (1.3x1.3x4) mm^3^ tended to overestimate RV volumes compared to SSFP at 1.5T (mean difference of RVEDV 8.2±9.3ml) and to FGRE at 7T with voxel size (1.2x1.2x6) mm^3^ (mean difference of RVEDV 9.3±8.6ml).

**Conclusions:**

FGRE cine imaging of the RV at 7T was feasible and provided good image quality. RV dimensions and function were comparable to SSFP at 1.5T as gold standard.

## Background

Function, size and morphology of the right cardiac ventricle (RV) are known to be strong influencing factors on morbidity and mortality in various cardiac diseases, for instance congenital heart disease, pulmonary hypertension, myocardial infarction or arrythmogenic right ventricular cardiomyopathy (ARVC) [1-4]. However, their evaluation by using non-invasive imaging techniques is often a challenge, mainly attributable to the asymmetric and highly variable shape of the RV, the predominantly longitudinal systolic shortening, the small myocardial wall thickness and the location behind the sternum. Cardiovascular magnetic resonance (CMR), mainly using steady-state free-precession (SSFP) cine imaging at a field strength of 1.5Tesla (T), has evolved as the gold standard for the assessment of RV dimensions and function due to its ability to image in any plane, its excellent blood-tissue contrast, its capability to depict even small wall motion abnormalities and its proven reproducibility [4-7]. Nevertheless, expectations to characterize the myocardial tissue of the RV comparable to the LV including the differentiation of fibrosis, fat or edema, remained widely unaccomplished yet. As an example, the current task force criteria to assess ARVC include CMR to assess RV size and function, but emphasize that the evidence of fibro-fatty replacement within the RV wall is only obtained by endomyocardial biopsy [4]. Even the accuracy of CMR to quantify RV mass, which is known to be an important predictor of cardiovascular events, is uncertain due to the challenge of resolving the thin (2-5mm) RV free wall properly using protocols (typical voxel size of cine imaging 1.8x1.8x6mm^3^) common in today’s clinical [8-10]. To extend the information that is extractable from CMR, technological improvements that increase the spatial and temporal resolution as well as signal-to-noise ratio (SNR) are therefore desired. As field strength positively correlates with SNR, CMR at ultrahigh fields (7T) offers the potential to depict even microscopic structures and to facilitate targeted tissue characterization [11,12]. However, imaging at 7T comes with technical challenges, like increased B_0_ heterogeneities, non-uniform B_1_ distribution and increased radio frequency (RF) power deposition. Recently, the technical feasibility of cardiac cine imaging at 7T using fast gradient echo (FGRE) techniques has been demonstrated, and data demonstrating the ability for accurate LV chamber quantification at 7T were reported [13-16]. Moreover, dedicated transmit and receive coils as well as cardiac trigger techniques have been developed to meet the demands of CMR at 7T [17-21].

Aim of the present study was to extend the application of cine imaging at 7T to the assessment of RV size and function and to compare the results with the gold standard at 1.5T. This work is regarded as the first step toward a comprehensive assessment of RV function, size and morphology using CMR at 7T.

## Methods

### Subjects

Nine healthy volunteers (6 males; mean age 29±5 years, range 24–38 years; mean body mass index 22±2 kg/m^2^, range 18-24kg/m^2^) underwent CMR at 1.5T and at 7T. The status “healthy” was based on: i) uneventful medical history, ii) absence of any symptoms indicating cardiovascular dysfunction, iii) normal ECG, iv) normal cardiac dimensions and function on the CMR cine images of this study. The local ethics committee approved the study, and all subjects gave written informed consent to participate in the study.

### MR and RF coil technology at 7T

A 7T whole body MR system (Siemens Healthcare, Erlangen, Germany) equipped with an Avanto (Siemens Healthcare, Erlangen, Germany) gradient system (slew rate: 200 T/m/s, max. gradient strength: 40mT/m) and an 8kW RF amplifier (Stolberg HF-Technik AG, Stolberg-Vicht, Germany) was used. A 16 channel transceiver coil array tailored for cardiac MR at 7.0 T [19,20] that uses loop elements and consists of two sections was applied: A planar posterior section located below the patient’s chest and a modestly curved anterior section positioned on top of the patient’s chest. Each section contains an identical arrangement of 8 TX/RX elements, connected to the MR system via a coil interface comprising 16 TX/RX switches and low-noise preamplifiers (Stark Contrasts, Erlangen, Germany). To drive each channel of the coil the output of the RF amplifier was split into 16 equal-intensity signals using a home-built 16x16 Butler matrix. The transmit phases of the individual coil elements were adjusted by using the 1st order circular polarized (cp) mode of the Butler matrix with an increment of 22.5 degrees between subsequent output channels in conjunction with -78° phase shifting coaxial cables for all posterior coil elements [20].

### MR and RF coil technology at 1.5T

At 1.5T a Magnetom Avanto (Siemens Healthcare, Erlangen, Germany) system equipped with the same gradient coil used at 7.0 T was used. A TX body coil was used for transmission and a 12-channel RX body array coil was used for reception.

### Cardiac gating

Since electrocardiography is corrupted by interference with electromagnetic fields and by magneto-hydrodynamic effects at 7T, acoustic cardiac triggering (ACT) was applied (EasyACT, MRI.TOOLS GmbH, Berlin, Germany). ACT registers the first heart tone of the phonocardiogram to synchronize data acquisition with cardiac motion [21]. ACT was used for retrospective gating at 7T and at 1.5T to ensure consistency throughout the study.

### Slice positioning

One technician with profound experience performed all scans at 1.5T and 7.0T to ensure reproducibility and consistency. After acquisition of scout images, standard four- and two-chamber views as well as a long axis view of the RV were acquired. Next, a stack of transverse slices covering the entire RV was obtained twice using two voxel dimensions. These images served for RV volumetry and evaluation of image quality. Furthermore, a stack of sagittal slices covering the entire RV was acquired. These images only served for evaluation of image quality.

### Imaging parameters

At 7T, cine images were acquired using 2D FGRE. Each slice was acquired in an end-expiratory breath-hold. Imaging parameters were: echo time TE=2.7ms, repetition time TR=5.6ms, nominal flip angle FA=32°, transmit reference voltage U_ref_ 400 V, peak voltage of sinc-pulse (t=800 μs) U_peak_ 190V, voxel size (1.2x1.2x6)mm^3^, no interslice gap, bandwidth 444Hz/pixel, 30 phases per heart cycle, parallel imaging (R=2, 32 calibration lines) in conjunction with GRAPPA reconstruction. For the transverse stack a second scan was performed using the same imaging parameters with the exception of the spatial resolution, which was adjusted to a voxel size of (1.3x1.3x4)mm^3^. Prior to RV function assessment volume selective second order B_0_-shimming was performed.

At 1.5T, a clinically established SSFP cine protocol was used. Imaging parameters were: TE=1.3ms, TR=3.0ms, FA=70°, voxel size (1.2x1.2x6)mm^3^, no interslice gap, bandwidth 790Hz/pixel, 30 phases per heart cycle, parallel imaging (R=2, 32 calibration lines) in conjunction with GRAPPA reconstruction.

### Image interpretation

The CMR images were read in consensus by two physicians (FvKB, VT) with very profound experience in CMR both at 7T and 1.5T [14,19,7]. The software CMR42® (Circle Cardiovascular Imaging, Calgary, Canada) was used. *Quantitatively*, RV end-diastolic volume (RVEDV), RV end-systolic volume (RVESV), RV ejection fraction (RVEF) and RV mass (RVM) were obtained by manually contouring endocardial borders in end-diastole and end-systole as well as epicardial borders in end-diastole. *Qualitatively*, each cine slice was scored in diastole and systole regarding overall image quality, homogeneity and contrast. The decision was based on anatomic border sharpness, visualization of subtle anatomic features and presence of artifacts. i) Overall image quality: 0=poor, non-diagnostic; 1=impaired image quality that may lead to misdiagnosis; 2=good; and 3=excellent. ii) Homogeneity: 0=inhomogeneous image or artifacts that may impair diagnostic quality; 1=homogeneous image, no artifact present. iii) Image contrast: 0=low or poor contrast between anatomical structures and blood pool; 1=sufficient contrast between anatomical structures and blood pool.

### Statistical analysis

Continuous values (RVEDV, RVESV, RVEF, RVM) are displayed as median and range. Kruskal-Wallis tests were performed to evaluate differences between CMR settings (1.5T vs. 7T with voxel size (1.2x1.2x6)mm^3^ vs. 7T with voxel size (1.3x1.3x4)mm^3^). Bland-Altman analyses were performed to assess the pairwise agreement. For categorical data (image quality, contrast, homogeneity), relative frequencies are shown. Multinomial or logistic regression was used to evaluate differences between the different CMR settings. The regression analyses were based on generalized estimation equations with independence as working correlation matrix to account for multiple measurements per patient. A p-value of less than 0.05 was regarded as statistically significant. Calculations were done with SAS 9.2 (SAS Institute Inc., Cary, NC, USA).

## Results

### Evaluation of overall image quality, image contrast and homogeneity

**Figure 1:**
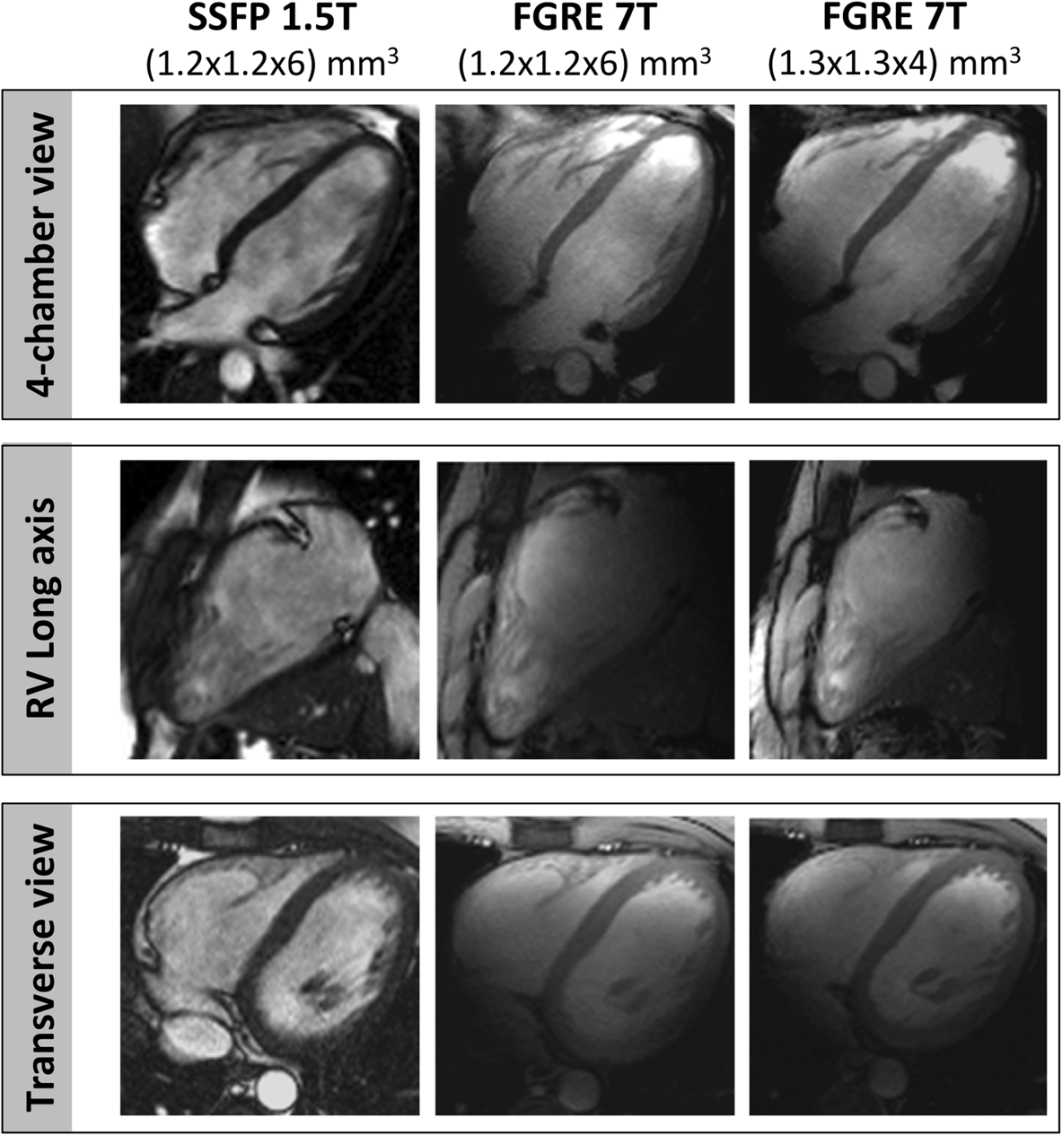
**Cine imaging of the RV. **Four-chamber view, RV long axis view and mid-ventricular transverse view obtained by SSFP at 1.5T with voxel size (1.2x1.2x6)mm^3^*(left)*, by FGRE at 7T with voxel size (1.2x1.2x6)mm^3^*(middle) *and by FGRE at 7T with voxel size (1.3x1.3x4)mm^3^*(right)*.

**Figure 2:**
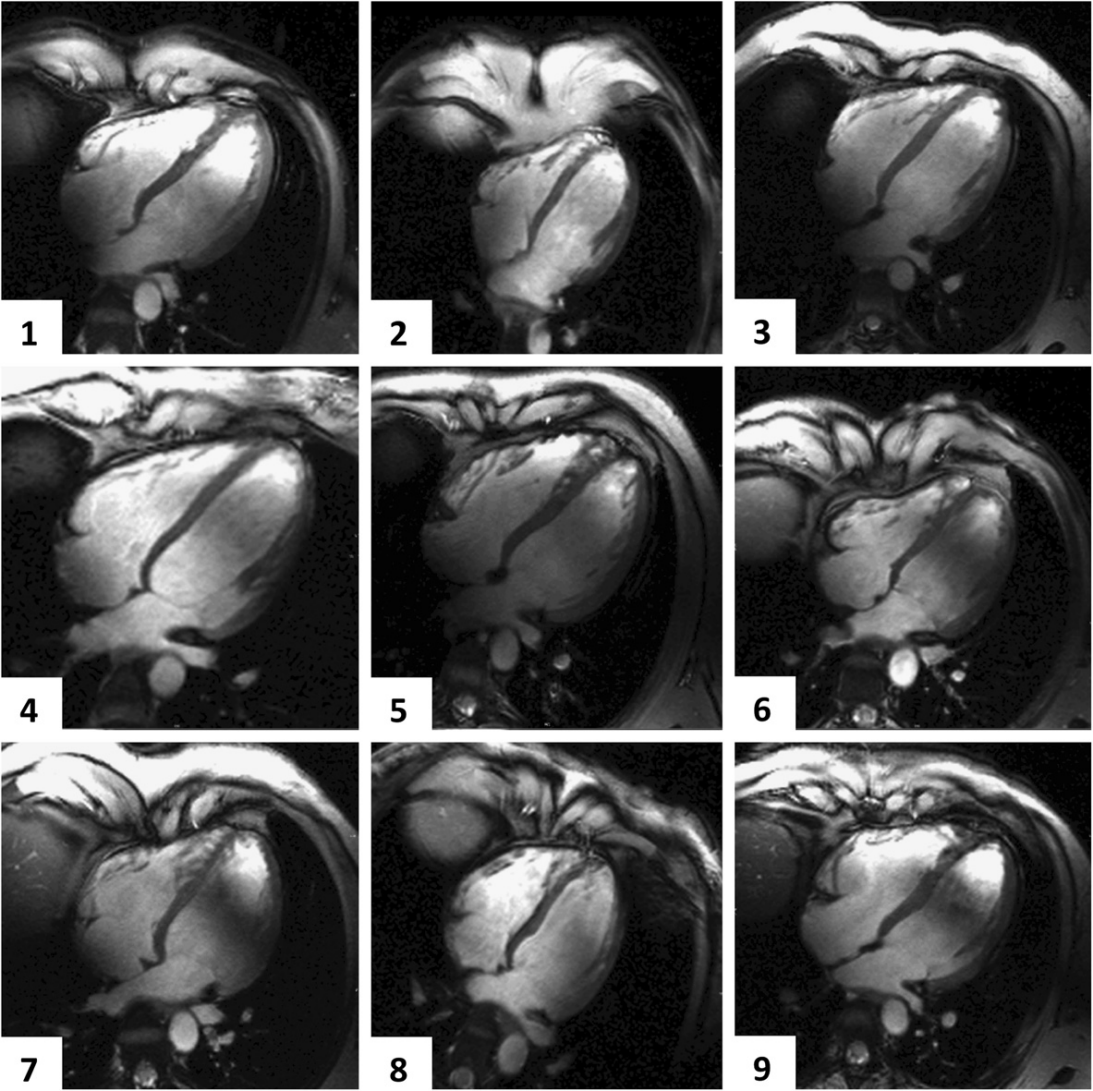
**Cine imaging of the RV. **Four-chamber views of all subjects obtained with 2D cine FGRE at 7T using a voxel size of (1.3x1.3x4) mm^3^.

Image quality was diagnostic in all CMR examinations at 1.5T and 7T, with no examination scored as “poor / non-diagnostic” (score 0). Figure 1 exemplarily depicts a four-chamber view, a RV long axis view and a mid-ventricular transverse slice of the RV obtained with FGRE at 7T together with SSFP images derived from 1.5T. Figure 2 provides an overview of the four-chamber views of all subjects derived from 7T. Additional movie files show cines of the RV long axis and the transverse slice acquired at 7T in one subject, and a composition of the four-chamber views of all study participants obtained at 7T (see Additional files 1, 2 and 3).

**Figure 3:**
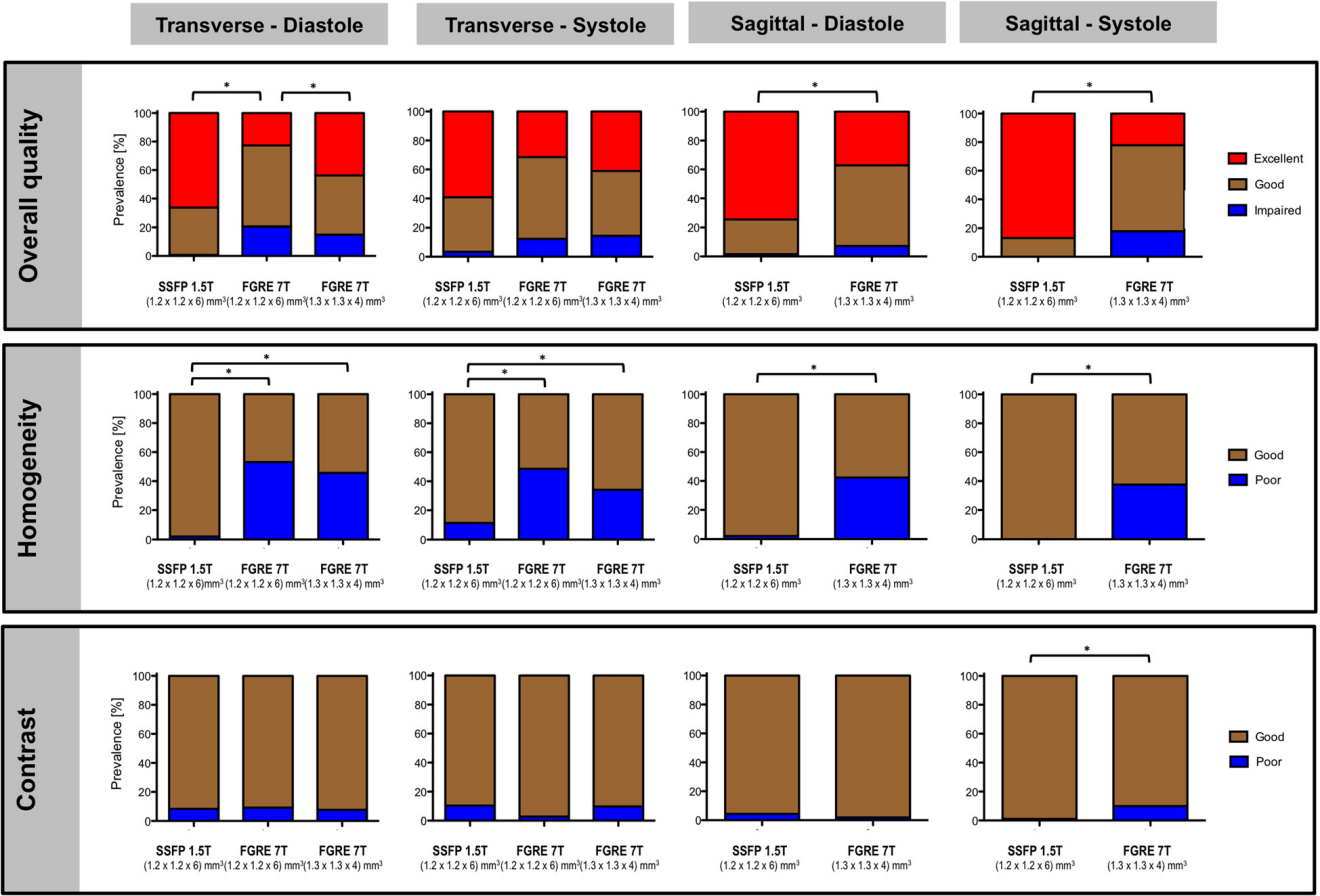
**Image quality, contrast and homogeneity. **Scoring results are grouped by slice orientation (transverse and sagittal) and by cardiac phase (diastole and systole). Significant inter-group differences are highlighted by “*”. The corresponding p-values are given in the text.

The results of the evaluation of overall image quality, image contrast and homogeneity are summarized in Figure 3:

Regarding *overall image quality*, SSFP at 1.5T and FGRE at 7T with voxel size (1.3x1.3x4)mm^3^ provided a significantly higher proportion of “excellent image quality” (66.4% and 43.8%) compared to FGRE at 7T with voxel size (1.2x1.2x6)mm^3^ (22.7%) in the transverse slice orientation in diastole (p<0.0001 and p=0.0478). For the sagittal slice orientation, SSFP cine images at 1.5T provided a significantly higher proportion of “excellent image quality” (75% in diastole and 87% in systole) compared to FGRE at 7T with voxel size (1.3x1.3x4)mm^3^(37% in diastole and 22% in systole) (each p<0.0001).

Regarding *image homogeneity*, SSFP at 1.5T provided significantly higher proportions of “homogeneous images” compared to the 7T acquisitions, both in transverse orientation in diastole (p=0.0329) and in systole (p=0.0197), and in sagittal orientation in diastole (p=0.0002) and systole (p<0.0001). The 7T groups did not differ significantly from each other.

Regarding *image contrast*, the various modalities were not found to differ significantly in transverse image orientation (diastole: p=0.7290; systole: p=0.3170), as well as in sagittal image orientation in diastole (p=0.1182). Only for systole in sagittal orientation, SSFP at 1.5T provided a higher proportion of images rated as “sufficient contrast” compared to FGRE at 7T with voxel size (1.3x1.3x4)mm^3^ (p=0.0463).

### RV chamber quantification

**Figure 4:**
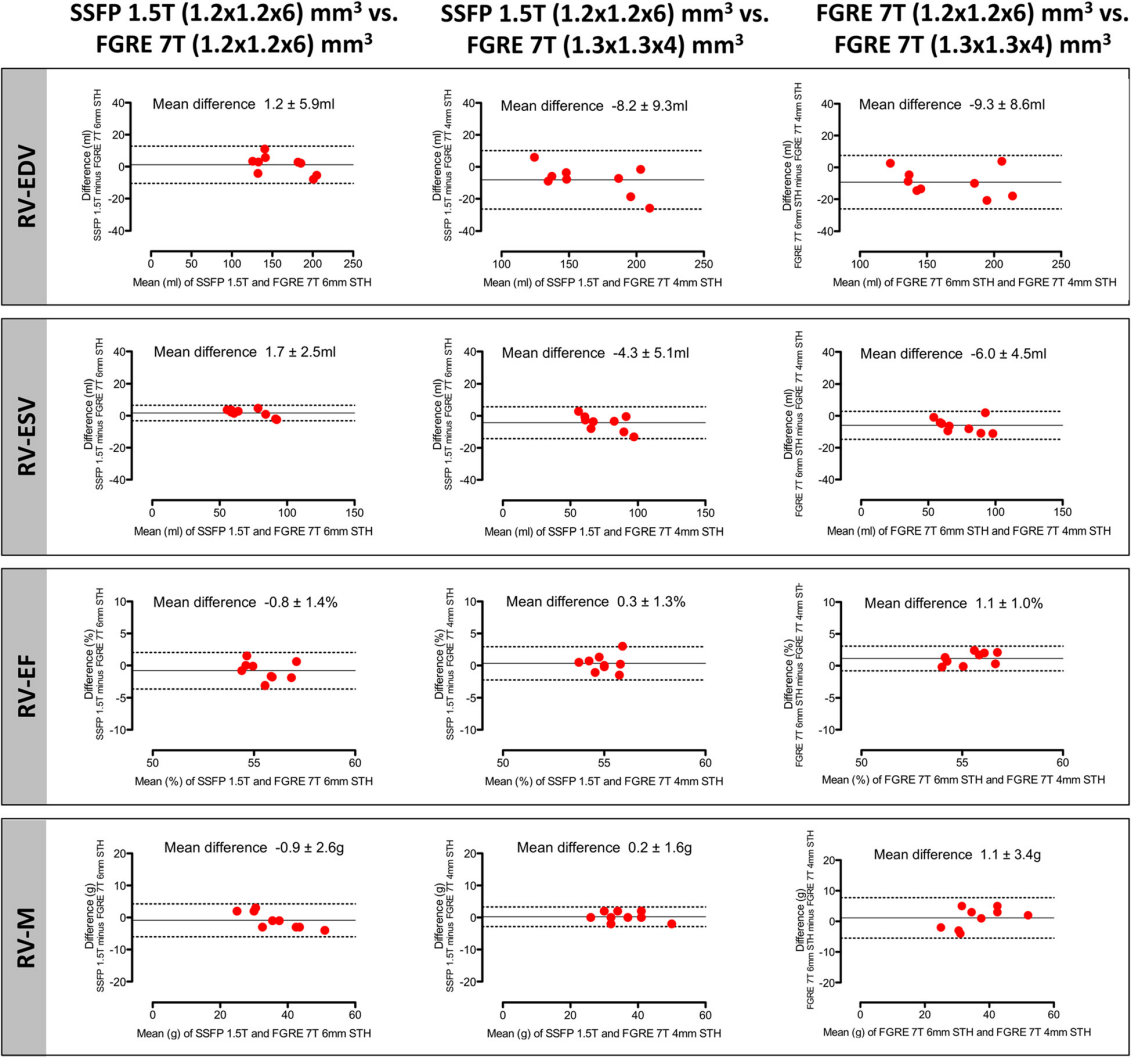
**RV chamber quantification. **Bland-Altman plots illustrating the agreement between RV volumes, ejection fraction and mass obtained by SSFP at 1.5T as the current gold standard and those derived from FGRE cine acquisitions with different spatial resolutions. [RV-EDV = right ventricular end-diastolic volume; RVESV = right ventricular end-systolic volume; RVEF = right ventricular ejection fraction; RVM = right ventricular mass].

Median quantitative RV results are depicted in Table 1. No significant difference was found regarding RVEDV (p=0.5850), RVESV (p=0.5462), RVEF (p=0.2789) and RVM (p=0.0743) between SSFP at 1.5T and FGRE at 7T. Bland-Altman plots illustrating the agreement between the various approaches regarding REDV, RVESV, RVEF and RVM are shown in Figure 4. There was a tendency towards larger volumes with FGRE at 7T with voxel size (1.3x1.3x4)mm^3^ both compared to SSFP at 1.5T (mean difference: RVEDV -8.2±9.3ml, RVESV -4.3±5.1ml) and FGRE at 7T with voxel size (1.2x1.2x6)mm^3^ (mean difference: RVEDV -9.3±8.6ml, RVESV -6.0±4.5ml).

**Table 1:** RV chamber quantification

	**1.5T SSFP 1.2x1.2x6mm**^**3**^	**7T FGRE 1.2x1.2x6mm**^**3**^	**7T FGRE 1.3x1.3x4mm**^**3**^	**p-value**
**RVEDV [ml]**				0.5850
Median	146.2	138.6	152.1	
Min. - Max.	127.4 - 202.3	124-0 - 207.7	121.4 - 222.8	
**RVESV [ml]**				0.5462
Median	65.2	62.4	69.3	
Min. - Max.	57.4 - 91.1	53.7 - 93.5	54.6 - 103.6	
**RVEF [%]**				0.2789
Median	55.0	56.7	55	
Min. - Max.	54 - 57	54 - 58	54 - 58	
**RVM [g]**				0.0743
Median	35	36	33	
Min. - Max.	26 - 49	24 - 53	26 - 51	

Legend: RV end-diastolic volume (RVEDV), RV end-systolic volume (RVESV), RV ejection fraction (RVEF) and RV mass (RVM) are presented as median, min and max for each modality (1.5T SSFP, 7T FGRE with two voxel sizes). The p-values refer to the Kruskal-Wallis test including all three modalities. There was no significant difference regarding RVEDV, RVESV, RVEF and RVM.

## Discussion

The present results demonstrated that FGRE cine imaging of the RV at 7T using a dedicated 16-channel transceiver RF coil and acoustic cardiac gating provided good quality cine images of the RV to assess its size and function. The findings confirm preliminary experiences with the LV at 7T [14] and underline that cine imaging at 7T is feasible.

We know from studies of the LV with FGRE cine imaging at 1.5T and 7T that the step from 1.5T towards 7T improved the image quality of FGRE cine images, in particular in long axis acquisitions parallel to the blood flow [14]. The blood/myocardium contrast is known to be reduced with low blood flow, attributable to the inflow-dependent blood pool signal intensity in FGRE sequences [22]. The ultrahigh field strength has the potential to minimize this source of error due to different field strength dependent T_1_ prolongation of blood and myocardium. Translated to the RV, this observation gains importance due to sometimes low blood flow between the RV trabeculae. In the present study of the RV, images in transverse orientation acquired with SSFP at 1.5T received the highest mean image score compared to the acquisitions at 7T. Notwithstanding, it is notable that our statistical analysis revealed that both field strengths did not differ significantly in overall image quality in systole in general, as well as in diastole if the enhanced spatial resolution (voxel size (1.3x1.3x4)mm^3^) is regarded. This observation underlines the feasibility to obtain high-quality cine images of standard planes at 7T and forms the basement for accurate RV chamber quantification, which is predominantly done in transverse slices [6]. In contrast, the sagittal orientated slices exhibited significant differences in image quality between SSFP at 1.5T and FGRE at 7T, with SSFP at 1.5T being more often “excellent”. The observation that the image quality at 7T varied dependent on slice orientation can be attributed to the complex B_1_ field pattern with various constructive and destructive interferences throughout the FOV. Depending on the volunteer and slice orientation, a specific field pattern is revealed. From a clinical perspective, sagittal views are currently mainly used to assess RV wall motion and morphology complementary to transverse slices. This comprehensive assessment is important for example in the diagnosis of ARVC, where regional akinesia, dyskinesia or asynchrony are relevant indicators [4]. Therefore, further technical improvements are necessary to enable patient and slice dependent transmit field shaping to provide stable image quality throughout a CMR examination at 7T.

It is a recognized limitation of this study that no SNR and blood/myocardium contrast-to-noise ratio (CNR) values are reported. A previous pilot study on cardiac chamber quantification at 1.5 T and 7.0 T showed that 2D cine FGRE imaging at 7.0 T provided SNR and blood/myocardium CNR that are superior to FGRE acquisitions at 1.5T using the same slice thickness [14]. Careful SNR and CNR validation demonstrated that SNR and CNR derived from 2D cine FGRE at 7T was found to be competitive with that derived from SSFP acquisitions at 1.5 T using standard clinical routine imaging parameter [14].

Regarding image contrast as assessed by visual evaluation, the present results underline that the combination of FGRE with ultrahigh field reaches a grade of contrast that is comparable to SSFP at 1.5T, similar to the results reported for the LV [14]. For the RV, this was true both for transverse and sagittal slice orientation and independent from the voxel size. Opposed to that, the acquisition of homogeneous images is still a challenge in ultrahigh field CMR. Both in sagittal and transverse direction, the results at 7T were inferior compared to 1.5T and a high proportion of 7T images were evident with poor homogeneity for both voxel sizes. Further improvements in subject specific and slice specific B_0_- and B_1_-shimming are therefore essential to fully exploit the potential of 7T [23,24].

**Figure 5:**
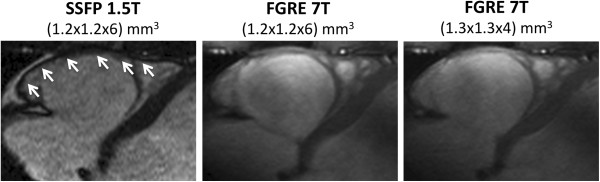
**Blood-myocardium contrast of the RV free wall. **Focused view on the RV free wall demonstrating that the border between blood and myocardium appears less dark and less sharp at 7T compared to 1.5T, which is further enhanced with decreasing voxel size. This potentially explains the tendency towards larger volume measurements at 7T with voxel size (1.3x1.3x4)mm^3 ^compared to 1.5T.

Regarding the quantification of cardiac size and function, it has been shown for 1.5T and 3T that RV volumes are overestimated and mass underestimated when using SSFP instead of FGRE [22,10] - even though the absolute difference is relatively small with a mean RVEDV difference of about 4ml [22]. This difference is explained by the stronger contrast between myocardium and blood on the one side, and between myocardium and epicardial fat on the other side obtained with SSFP. This leads to an outward shift of the endocardial contour and an inward shift of the epicardial contour compared to FGRE. Furthermore, areas with slow flow, as within the apex, are often mistaken for myocardium when using FGRE. It is notable from the present results that the difference in RV quantification between SSFP and FGRE no longer existed when moving from 1.5T to 7T. This tendency matches the experiences we made for the LV at 7T [14]. The bias between the results obtained for FGRE imaging at 7T with voxel size (1.2x1.2x6)mm^3^and SSFP at 1.5T (RVEDV 1.2±5.9ml, RVESV 1.7±2.5ml) was within the range of the inter-observer variability reported for SSFP examinations of the RV [22]. Hence, close agreement can be postulated. This finding underlines the accurate endocardial border delineation achievable by FGRE at 7T. Another interesting aspect of the present results was that FGRE at 7T by tendency provided larger volumes than SSFP 1.5T when we decreased the voxel size from (1.2x1.2x6)mm^3^ to (1.3x1.3x4)mm^3^. We explain this trend mainly by the observation that the higher spatial resolution led to a more detailed differentiation of the RV free wall that caused an outward shift of the manually contoured endocardial borders (Figure 5). This finding on the one hand has to be kept in mind when trying to translate normal values from 1.5T to 7T. On the other hand, it underscores the potential of CMR at 7T to have a more detailed look at the RV free wall. As it can be anticipated from Figure 3, some inter-subject variability of trabecular structure and compact layer thickness may exist. For instance, the measurement of the compact layer of the free RV wall as well of the remaining trabecular layer may provide new insights into the microstructure of the RV myocardium. Furthermore, the accurate quantification of RV mass based on properly spatially resolved cine images would strengthen its role as a cardiovascular risk marker. After this first evidence of the feasibility of RV cine imaging at 7T, such interesting aspects need further investigation in future studies with sample sizes large enough to provide adequate power.

## Conclusions

In conclusion, the present study demonstrated that cine imaging of the RV is feasible at 7T using FGRE techniques. The achieved image quality was comparable to SSFP at 1.5T and allowed accurate myocardial border delineation for RV chamber quantification, which provided RV volumes, mass and function with close agreement to SSFP at 1.5T. This work is regarded as fundament and stimulator for further efforts to explore whether enhanced spatial resolution together with ultrahigh field strength can provide more detailed insights into the structure of the RV.

## Competing interests

The authors declare that they have no competing interests with the exception of Thoralf Niendorf and Jan Rieger, who are founder of MRI.TOOLS GmbH (Berlin, Germany).

## Authors’ contributions

FvKB and VT contributed to the study design, image acquisition, image interpretation, data storage, statistical data analysis and data interpretation and drafted the first version of the manuscript; LW and CT contributed to the image acquisition (in particular regarding coils) and data interpretation; JR contributed to the image acquisition (in particular regarding cardiac gating) and data interpretation; MD contributed to the image acquisition, image interpretation and data interpretation; TN and JSM contributed to the study design, image acquisition, image interpretation, data storage, statistical data analysis and data interpretation. All authors helped to draft the manuscript, and read and approved the final manuscript.

## Supplementary Material

Additional file 1 **Long axis view of the right ventricle obtained by FGRE cine imaging at 7T with voxel size (1.3x1.3x4)mm**^**3 **^**underlining the excellent blood/myocardium contrast even in the right ventricular apex.** Click here for file

Additional file 2 **Transverse view of the right ventricle obtained by FGRE cine 7T with voxel size (1.3x1.3x4)mm**^**3 **^**underlining the excellent resolution of the RV trabeculae.** Click here for file

Additional file 3 **Cinematic four-chamber views of all subjects obtained with 2D cine FGRE at 7T using a voxel size of (1.3x1.3x4) mm**^**3**^**.** Click here for file
